# Incentivising public transport use for physical activity gain: process evaluation of the COVID-19 disrupted *trips4health* randomised controlled trial

**DOI:** 10.1186/s12966-022-01394-x

**Published:** 2022-12-22

**Authors:** K. A. Jose, M. J. Sharman, O. Stanesby, S. Greaves, A. J. Venn, L. Blizzard, A. Palmer, K. Cooper, J. Williams, V. J. Cleland

**Affiliations:** 1 Menzies Institute for Medical Research, University of Tasmania, Hobart, Tasmania Australia; 2 Institute of Transport and Logistic Studies, The University of Sydney, Butlin Avenue, Darlington, Camperdown, New South Wales 2006 Australia; 3 Metro Tasmania, 212 Main Road, Moonah, Hobart, Tasmania 7009 Australia; 4 Public Health Services, Department of Health, Tasmanian Government, 2/25 Argyle Street, Hobart, 7001 Australia

**Keywords:** Motivation, Preventive health services, Translational medical research, Disease outbreaks, Public-private sector partnerships, Transportation facilities, Behaviour and behaviour mechanisms, Exercise, Walking

## Abstract

**Background:**

Partnering with a public transport (PT) provider, state government, and local government, the single-blinded randomised controlled trial, *trips4health,* investigated the impact of PT use incentives on transport-related physical activity (PA) in Tasmania, Australia. The intervention involved 16-weeks of incentives (bus trip credits) for achieving weekly PT use targets, supported by weekly text messages. This study objective was to conduct a process evaluation of the COVID-19 disrupted *trips4health* study.

**Methods:**

The Medical Research Council UK’s framework for complex public health interventions guided the process evaluation. Participant reach, acceptability, fidelity and feasibility were evaluated. Administrative and post-intervention survey data were analysed descriptively. Semi-structured interviews with intervention participants (*n* = 7) and PT provider staff (*n* = 4) were analysed thematically.

**Results:**

Due to COVID-19, *trips4health* was placed on hold (March 2020) then stopped (May 2020) as social restrictions impacted PT use. At study cessation, 116 participants (approximately one third of target sample) had completed baseline measures, 110 were randomised, and 64 (*n* = 29 in the intervention group; *n* = 35 in the control group) completed post-intervention measures. Participants were 18 – 80 years (average 44.5 years) with females (69%) and those with tertiary education (55%) over-represented. The intervention was delivered with high fidelity with 96% of bus trip credits and 99% of behavioural text messages sent as intended. Interviewed PT staff said implementation was highly feasible. Intervention participant acceptability was high with 90% reporting bus trip incentives were helpful and 59% reporting the incentives motivated them to use PT more. From a total of 666 possible bus trip targets, 56% were met with 38% of intervention participants agreeing and 41% disagreeing that ‘Meeting the bus trip targets was easy’. Interviews and open-ended survey responses from intervention participants revealed incentives motivated bus use but social (e.g., household member commitments) and systemic (e.g., bus availability) factors made meeting bus trip targets challenging.

**Conclusions:**

*trips4health* demonstrated good acceptability and strong fidelity and feasibility. Future intervention studies incentivising PT use will need to ensure a broader demographic is reached and include more supports to meet PT targets.

**Trial registration:**

ACTRN12619001136190.

**Supplementary Information:**

The online version contains supplementary material available at 10.1186/s12966-022-01394-x.

## Background

Physical inactivity is one of the most significant global health concerns, linked to 10 – 20% coronary heart disease, type 2 diabetes and breast and colon cancers [1]. Globally, physical inactivity was estimated in 2018 to cost the health care system INT$54 billion [2]. Despite extensive efforts over recent decades to increase leisure-time physical activity (PA) adherence to PA guidelines in Australia has stagnated since the 1980s, at around 35-40% [3]. Transport-related PA has been identified as a potential mechanism of increasing PA with studies showing that people who walk to public transport accumulate a meaningful amount of PA, contributing to the attainment of physical activity recommendations [4–7]. Transport-related PA provides an under-explored opportunity to increase PA whilst also leading to other positive outcomes such as lessening traffic congestion, environmental pollution and climate change [8]. Incentives-based strategies have shown promise for increasing PA [9, 10], but the impact of incentives on transport-related PA is unknown.

To fill an evidence gap, the *trips4health* single-blinded parallel design randomised controlled trial (RCT) investigated the impact of incentivising public transport use on transport-related PA. *trips4health* was undertaken in partnership with Australian researchers and policy makers in transport management, active living and public health and a local public transport provider. It was hypothesised that incentivising public transport use would result in an increase in public transport use leading to an increase in transport-related PA with subsequent health and wellbeing gains. The trial was abandoned in May 2020 due to the COVID-19 pandemic, but analysis of available outcome data showed average weekly bus use was higher for those in the intervention compared to the control group (2.5 bus trips compared to 1.8) (*paper under review*).

The study objective was to conduct a process evaluation of *trips4health.* Process evaluations of RCTs can determine fidelity and quality of implementation as well as clarify causal mechanisms and contextual factors associated with outcome variation [11]. Guided by the Medical Research Council UK’s framework for complex public health interventions [12], this paper focuses on implementation fidelity, feasibility, reach and acceptability of *trips4health*.

## Methods

### trips4health

*trips4health* was a RCT implemented within the Greater Hobart area (Hobart is the state capital of Tasmania). Tasmania is a regional island state of Australia with approximately 510,000 residents of which just under half lived in the study area [13]. The only mode of public transport during the study period was bus, with metropolitan services predominantly offered by one provider. In 2016, an estimated 5% of employed residents within the Greater Hobart area commuted by bus as a single method of transport and 76% by driving a car [14].

Full details of *trips4health* are outlined in the protocol [15] and are summarised here. *trips4health* was a RCT with a 16-week intervention phase and a 6-month follow-up phase targeting infrequent adult bus users. Eligible participants were randomised to an intervention or control group with participants in the intervention group rewarded with bus trip credits if they achieved weekly bus trip targets. Bus trip targets escalated over the course of the intervention with a subsequent increase in value of incentives. If the weekly bus trip target was met (confirmed through objective bus use smartcard data), participants received bus trip credit (through their smartcard, administered by the public transport provider). Participants aimed to achieve 5 one-way bus trips per week by the end of the intervention. Incentives were supported by other theory-informed behaviour change techniques (e.g., information on consequences of PA behaviour to the individual such as lowering the risk of diabetes, setting graded tasks and goal setting), delivered via weekly mobile phone text messages [16]. Both control and intervention group participants received printed versions of Australia’s Physical Activity and Sedentary Behaviour Guidelines [17] and up to $30 in smartcard credit for participating in the study (compensation). The primary outcome of *trips4health* was change in average daily step count measured by accelerometer (Actigraph GT3X). Secondary outcomes included change in travel behaviour and commute times, perspectives on travel behaviour, transport-related expenses, health-related physical measures (e.g., blood pressure) and quality of life. The logic model (Additional File Fig. S1) demonstrates the hypothesised causal mechanisms. The study was approved by the Tasmanian Health and Medical Human Research Ethics Committee on the 27 May 2019 (H0017820). All participants provided written or verbal consent to participate in the study. *trips4health* was registered with the Australian and New Zealand Clinical Trials Registry on the 14 August 2019 (ACTRN12619001136190).

### *trips4health* and the COVID-19 pandemic

**Fig. 1:**
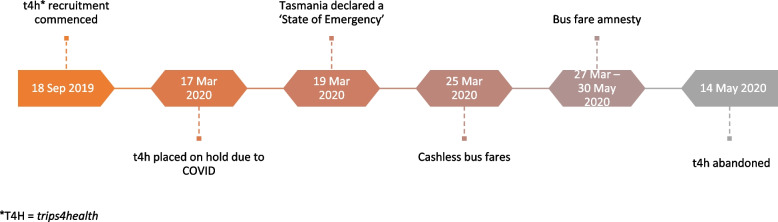
Timeline *trips4health* and Covid related public transport related changes

*trips4health* recruitment commenced on September 18, 2019, and continued until March 17, 2020 when investigators placed recruitment on hold in response to the COVID-19 pandemic reaching Tasmania. On March 19, 2020, the Tasmanian Government declared a state of emergency and social restrictions were implemented. Public transport use declined when Tasmanians were asked to stay at home and schools moved to online learning. From March 25, 2020, cashless bus fares were introduced, and a bus fare amnesty was in place from 27 March 27, 2020, to the May 31, 2020. Study investigators stopped *trips4health* on May 14, 2020, because of the broadscale social changes imposed by COVID-19, uncertainty about the progress of COVID-19, participant safety concerns from use of public transport, and the impact of COVID-19 on study validity because some participants would be entering the intervention phase (incentivising public transport use) of *trips4health* in a COVID-19 environment when others had not (Fig. 1).

**Fig. 2:**
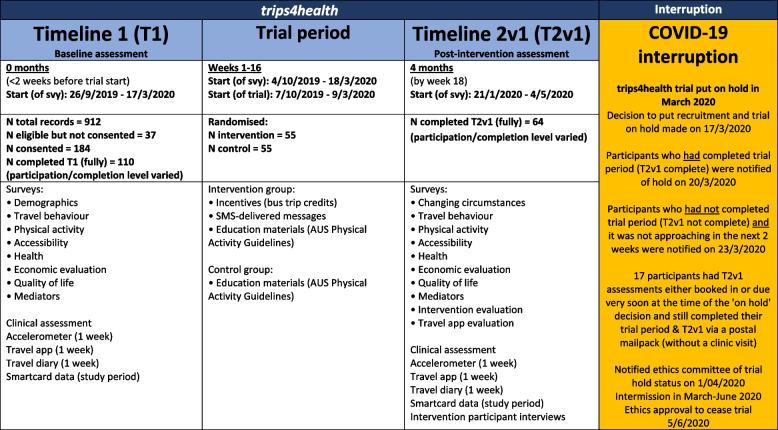
trips4health trial stages and participation levels

**Fig. 3:**
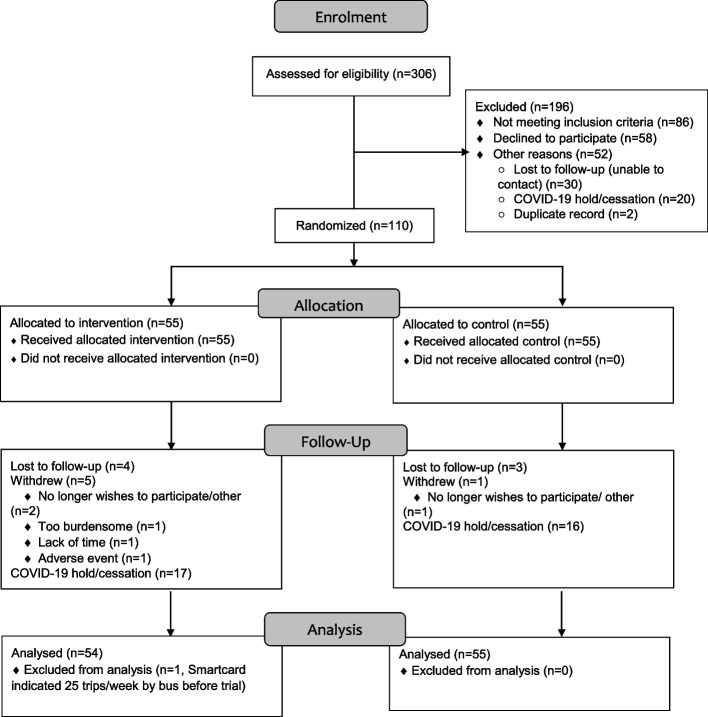
CONSORT flow diagram of participation

When *trips4health* was stopped, 110 participants had been recruited, undergone randomisation and completed baseline (Timepoint 1) assessments (Fig. 2 and Fig. 3). Of these, 64 had completed the intervention phase of *trips4health* and completed post-intervention (Timepoint 2) assessments (noting one control group participant submitted an incomplete survey). No participants had completed the 6-month maintenance phase and therefore there was no data collected at Timepoint 3. The depth and breadth of process evaluation data available at study cessation was considered sufficient to provide insights into *trips4health*’s implementation.

### Process evaluation data collection procedures and instruments

Surveys, interviews, administrative and travel smartcard (“Greencard” – used for local public transport trips) data were used to examine the implementation of *trips4health* (Table 1).

**Table 1:** Summary of data collection and number of participants

Process evaluation component	Data collection method				Number of participants			
	Administrative data	Survey data	Clinic assessments	Interview data	Administrative data	Survey data	Clinic assessments	Interview data *
Pilot	X	X	X	X	11	11	11^†^	6
Recruitment	X	X	–	–	912 (enquiries)	912 (enquiries)	–	–
Eligibility	X	X^‡^	–	–	306 (completed)	306 (completed)	–	–
Audit	X	X	X	–	30	30	30	–
Intervention fidelity (control group)	X	X	X	–	55	55 at T1, 35 at T2	55 at T1, 23 at T2	–
Intervention fidelity (intervention group)	X	X	X	X	55	55 at T1, 29 at T2	55 at T1, 23 at T2	11
Intervention acceptability (control group)	X	X	X	–	55	55 at T1, 35 at T2	55 at T1, 23 at T2	–
Intervention acceptability (intervention group)	X	X	X	X	55	55 at T1, 29 at T2	55 at T1, 23 at T2	11
Intervention acceptability* (public transport provider)	–	–	–	X	–	–	–	4

* Public transport provider employees interviewed were asked about their experience of the pilot study † participants provided with an accelerometer and app instructions only ‡ Screening survey

#### Recruitment and eligibility for trips4health

Recruitment was through social and local radio and printed media, on-bus and bus exchange advertising, concurrent University of Tasmania travel survey and staff intranet, newsletters of relevant organisation such as state government, and personal and professional networks of the project team. Potential *trips4health* participants were screened for eligibility via an online or telephone questionnaire (Additional File Table A1) [15]. Key eligibility criteria included residing in the Greater Hobart area, being an adult (age ≥ 18 years), using the bus less than or equal to two trips per week in the past 6 months, and possessing or willingness to obtain a travel smartcard. Potential participants were asked where they had heard about *trips4health*.

#### Surveys

At Timepoint 1 and Timepoint 2 participants completed a survey that included demographic, travel, PA, health, and economic questions. General questions about *trips4health* such as motivation to participate, recruitment and provision of study information were completed by control and intervention participants at Timepoint 2. Intervention group participants were asked questions about the acceptability and perceived impact of the incentives and text messages with Likert scale responses (e.g., I found the Greencard credit incentives helpful – strongly agree, agree, neither agree nor disagree, disagree, strongly disagree). Several open-ended response questions were included (e.g., What have you liked most about this study so far?).

#### Administrative data

The public transport provider smartcard data system captured date, time and fare type information for trip commencement for all bus users. The incentive (i.e., smartcard credit) was commensurate with the participants usual fare type (e.g., adult [AUS$3.50–7.20] and concession [AUS$2.80–5.76]) and was credited directly to the participants’ smartcard at the end of the week, contingent on smartcard data indicating that they had reached their weekly bus trip target. Weekly reports of participants’ smartcard data were provided to the research team by the public transport provider. The research team established whether targets had been met, and the public transport provider allocated credits. Participants were emailed weekly (mid-week) with information about whether they had reached their weekly target and the following week’s target. Targets increased as per the pre-determined schedule irrespective of reaching previous targets (Additional File Table A2).

Weekly mobile phone text messages were used to support behaviour change. Messages were matched to recognised behavioural change techniques such as rewards, goals and planning and self-belief and linked to the stage of the intervention but not tailored for individuals [16]. For example, an early message was, *Setting small goals can help motivate you. Why not try setting a goal to catch the bus 2 times more than usual this week?* A later message was, *Don’t let bad weather stop you from taking the bus and being more active - rug up, wear a jacket and grab your brolly!* (Additional File Table A3). Written materials (Australia’s Physical Activity and Sedentary Behaviour Guidelines) were provided at study commencement to all participants. Data on the number and timing of the weekly emails, text messages and provision of written materials were captured using administrative systems.

All eligible participants were scheduled to complete assessments at baseline (Timepoint 1), on completion of the 16-week intervention phase (Timepoint 2) and at the end of the six-month maintenance phase (Timepoint 3). A stepped approach to assessment participation (minimum, medium, high) was used to ensure a minimum amount of data (survey, smartcard, accelerometer) were collected from all participants with more burdensome measures (physical measures, travel behaviours via an app) related to secondary measures collected from those who were willing to do so. Participants nominated their preferred assessment participation level. Compensation (smartcard credit) for participation was stepped and linked to assessment completion at each timepoint but not to the level of assessment completed (AUD$5 at completion of Timepoint 1, AUD$10 at completion of Timepoint 2, AUD$15 at completion of Timepoint 3).

#### Interviews

Semi-structured interviews occurred with public transport provider employees with varied roles in the implementation of *trips4health* (marketing, research partnership contract, data systems) by author MS between August and November 2019 (*n* = 4). Timing of the interviews occurred pre or during *trips4health* recruitment as relevant to the role of the employee. The interviews focused on resources, process or policy changes (e.g., applying bus trip credits) relevant to implementation of *trips4health* and any factors that may impact implementation and benefits or anticipated benefits of *trips4health* once the findings of the study were known. Seven interviews were conducted with intervention group participants by a research assistant between May and June 2020 (after *trips4health* was placed on hold). These interviews focused on the conduct of *trips4health*, implementation processes and impact of the incentives and text messages (Additional File Table A4). Participants were selected from a sample of 15 who had completed the 16-week intervention phase and who had indicated in the Timepoint 2 survey their interest in being interviewed.

#### Pilot testing and audit

Between 27 June 2019 and 4 July 2019, 11 volunteers (as distinct from randomised participants) completed a two-week pilot testing phase. During this time, all *trips4health* processes and systems were checked, including connectivity between the *trips4health* administrative databases and public transport provider. All pilot testing volunteers were assigned to the intervention group and five completed a feedback survey. Additionally, for quality assurance purposes and to review *trips4health* processes an audit was conducted after the first 30 participants had been randomized.

### Analysis

Quantitative study data were collected and managed using REDCap electronic data capture tools hosted at University of Tasmania [18, 19]. Descriptive statistics (mean and standard deviation [SD], frequencies and percentages) were calculated using Stata [20] and Microsoft Excel® to describe the characteristics of the sample, recruitment data, acceptability measures and administrative data. All interviews were audio recorded, fully transcribed and de-identified. Transcripts and open-ended survey responses were imported into qualitative data analysis software NVivo 12 (QSR International) [21] before being read. Inductive coding was used to identify and categorise codes before identifying key themes. The analytic team (KJ, MS, VC) met to discuss coding decisions, emerging themes and refine the analysis. Criteria for coding was recorded within nodes in NVivo and coding decisions, key concepts, ideas, and reflections were identified and recorded in the project log and memos by KJ [22]. Quantitative and qualitative data was then synthesised [23]. All participant quotes are presented with an ID number, e.g., P16. Quotes from open-ended survey responses include an S, e.g., PS16 with public transport provider interviews using the prefix PP.

## Results

### Implementation Fidelity and feasibility

Pilot testing identified issues relating to communication clarity with participants, travel app usage and identifying participants who met bus trip targets. Subsequent modifications included improving study information provided to participants, offering a paper-based travel diary in addition to the travel app, refining the travel app and improving the reporting and recording of smartcard data (Additional File Table A5). The audit (conducted after the first 30 participants were randomised) revealed some inconsistencies in the consent process, miscalculation of bus trip targets and payment of incentives, travel app errors and confusion about assessment requirements. Refinements were made to address these problems once they were identified (Additional File Table A6).

All four public transport provider staff interviewed were positive about *trips4health* and viewed the research partnership as beneficial, *“…it’s really brilliant, usable, effective research, that’s done by a third party that people trust, that lends weight and credibility and credence to whatever the outcomes are*.” [PP1] Resource input was described as minimal, highlighting the potential scalability of the intervention, although scalability was flagged as potentially limited by state government contractual obligations dictating fleet size. Pilot testing was viewed as essential for ensuring that *trips4health* ran smoothly at a systems level.

The flow of participants through *trips4health* is described in Fig. 3. Of the 55 people randomised to the intervention group, 35 (64%) completed the 16-week intervention with 17 (31%) unable to complete the intervention phase because of COVID-19 related social restrictions. Post-intervention assessments were completed by 29 (83%) of the participants who completed the intervention. The vast majority (96%) of bus trip credits were delivered as intended, with missed credits later reimbursed to participants. For text messages, 99% of 1185 messages were sent as intended to intervention participants. Written materials were provided to all participants in the control and intervention groups either in person during assessments or via email (Table 2).

**Table 2:** Fidelity of key intervention components

Intervention components	Process Evaluation variables		
	Dose	Reach n (N)	Fidelity
Education materials*	Once	110/110	110/110
Incentives (smartcard credits – intervention group)	Weekly for 16-week intervention phase	35/55 35 completed 16- week intervention^**§**^	14 bus trip credits were missed and credited late. 3 smartcards replaced and updated for crediting. 1/3 missed an incentive because of card replacement that was later credited Intervention group *N* = 55: 375/666 possible bus trip targets met
Text messages (intervention group)	2 x p/w first 12 weeks, then 1 x p/w until end of 16-week intervention phase	55/55	1185 sent, 17 did not reach participants

* Australia’s Physical Activity and Sedentary Behaviour Guidelines

^**§**^Of the 35 who completed the 16-week intervention period 29 completed the follow-up assessment surveys

### Reach

Of the 912 people who enquired about *trips4health*, 444 (49%) indicated how they had found out about *trips4health*. Of the 444, bus advertising (36%) and social media (30%) were the most common mechanisms (Additional File Table A7). Of the 306 people who completed the eligibility assessment, the most common reasons for ineligibility were, that they were already catching the bus more than twice per week or that they were not making any trips by car that could be made by bus (Additional File Table A8). Of the 221 eligible participants interested in participating in *trips4health*, 184 (83%) consented to participate, with 110/184 (60%) completing all requirements to progress to randomisation. The most common reasons for not proceeding to randomisation were they were not contactable/no response (30/74; 41%), COVID-19 interruption (20/74; 27%) and personal or other reasons (12/74; 16%). Of the 110 participants randomised, participants’ age ranged from 18 to 80 years (average 44.5 years) with females (69%) and those with tertiary education (55%) over-represented compared to the Greater Hobart population (52% females and 17% tertiary educated) [13] (Table 3).

**Table 3:** Characteristics of participants

n	Randomised	Interviewees *
	110	7
**Age, mean (SD; range)**		
Years	44.5 (16.9; 18-80)	40 (13; 29-65)
**Sex, % (n)**		
Male	31 (34)	43 (3)
Female	69 (76)	57 (4)
**Education**^**§**^ **(highest), % (n)**		
Low	20 (22)	0 (0)
Medium	25 (27)	29 (2)
High	55 (61)	71 (5)
**Employment status, % (n)**		
Full-time	26 (29)	14 (1)
Part-time (20-34 hours/week)	21 (23)	29 (2)
Part-time (< 20 hours/week)	17 (19)	14 (1)
Retired	12 (13)	14 (1)
Not employed and not retired ^	15 (16)	0 (0)
Other or prefer not to answer	9 (10)	29 (2)
**Student status, % (n)**		
Yes	36 (40)	43 (3)
**Live with child/children under 15 years, % (n)**		
Yes ^#^	16 (18)	43 (3)

* Participant interviewees (intervention group participants only) in process evaluation. ^§^Education High: University degree or postgraduate qualification; Medium: Trade, apprenticeship, certificate, diploma: Low: Year 12 or less. ^ Not employed (looking for work), not employed (NOT looking for work), or not able to work; ^#^ Household is couple with children (under 15 years old) or one parent family

### Acceptability

Surveys completed at Timepoint 2 (*n* = 63, intervention group *n* = 29) and interviews conducted between 7 May and 11 June 2020 (*n* = 7) provided information about the acceptability of *trips4health*. Of all survey completers, 86% indicated it was easy/very easy to sign up for the study, 94% reported that the amount of information provided was ‘About right’ and 40% affirmed that the smartcard credit received as compensation motivated them to stay in the study.

For intervention group participants, 90% strongly agreed/agreed that bus trip credits were helpful, 97% strongly agreed/agreed that they liked the incentives, 90% strongly agreed/agreed that they received the incentives in an acceptable amount of time and 59% strongly agreed/agreed that the value of the incentives motivated them to use the bus more (Table 4).

**Table 4:** Acceptability survey responses

Participants and Trial component	Survey Questions	Strongly Agree/Agree	Neither Agree nor Disagree	Disagree/ Strongly Disagree
**All Participants**		**% (n)**	**% (n)**	**% (n)**
**Written Materials: Study information pamphlet (*n*** **= 63**^**†**^**)**				
I found this information about the study helpful		73 (46)	25 (16)	2 (1)
I found this information about the study easy to understand		76 (48)	22 (14)	2 (1)
Written Materials: Australia’s Physical Activity and Sedentary Guidelines (*n* = 63†)				
I found the information helpful *		59 (29)	41 (20)	0
I found the information interesting*		76 (37)	24 (12)	0
I liked this information*		57 (28)	43 (21)	0
This information made me think about increasing my physical activity levels*		45 (22)	41 (20)	14 (7)
This information made me increase my physical activity levels*		27 (13)	31 (15)	2 (1)
**Up to $30 Greencard credit for participation (*n*** **= 63)**				
The Greencard credit I received as compensation motivated me to stay in this study		40 (25)	38 (24)	22 (14)
I received the Greencard compensation credit in good time		83 (52)	14 (9)	3 (2)
**Intervention group only (*n*** **= 29)**				
**Incentives**				
I found the Greencard credit incentives helpful		90 (26)	7 (2)	3 (1)
The value of the Greencard credit incentives motivated me to use the bus more		59 (17)	28 (8)	14 (4)
I liked the Greencard credit incentives		97 (28)	3 (1)	0
The Greencard credit incentives had no impact on my bus use		10 (3)	31 (9)	59 (17)
The Greencard credit incentives had no impact on my physical activity		31 (9)	24 (7)	45 (13)
I received the Greencard credit incentives in an acceptable amount of time		90 (26)	10 (3)	0
Meeting the bus trip targets was easy		38 (11)	21 (6)	41 (12)
**Text Messages**				
The frequency of text messages was just right		76 (22)	17 (5)	7 (2)
The content of the text messages was easy to understand		90 (26)	7 (2)	3 (1)
I found the text messages helpful.		48 (14)	41 (12)	10 (3)
I found the text messages annoying		24 (7)	24 (7)	52 (15)
I found the text messages interesting		31 (9)	45 (13)	24 (7)
I found the text messages to be too long		0	38 (11)	62 (18)
I liked the text messages		28 (8)	59 (17)	14 (4)
**Weekly Emails**				
The weekly emails were helpful		59 (17)	35 (10)	7 (2)
I liked the weekly emails		59 (17)	28 (8)	14 (4)
The weekly emails made no difference to my bus use		45 (13)	14 (4)	41 (12)

^†^Of the 64 participants who completed the post-intervention assessments 1 control group participant did not complete the survey questions on acceptability. *Responses only for those who reported reading some (*n* = 18) or all (*n* = 31) of Australia’s Physical Activity and Sedentary Behaviour Guidelines *n* = 49

Interviewees and open-ended survey responses also indicated that participants liked the financial incentives and that the incentives motivated them to use the bus and overcome bus use barriers, as outlined by this participant:

*The incentive of having my trip paid for kind of got me over the high price of it. Because public transport isn’t always pleasant … But you just - you know, you see the benefit of the extra exercise, and then that incentive of having your trips paid for. Yeah, it definitely pushed me through the hard bits [P69].*

The value of the incentive was important for some people; *The incentive helped us catch the bus more. But the bus is generally very expensive per trip [P178].* However, the incentive value was not important for everyone; *It’s not really an incentive for me because as I say, I was under a concession scheme anyway [P413]. S*ome participants were motivated by being rewarded for reaching the bus trip target rather than by the incentive value, as this participant described:

*the way these incentives affected me it’s not the amount, but it’s the fact that whether you get it or not, whether you hit the target or not. Yeah. So it’s a motivating factor to hit the target. It’s like a reward; just the fact of it being a reward, not necessarily the amount of the reward [P247].*

In survey responses, 59% disagreed/strongly disagreed that the incentives had no impact on their bus use and 45% disagreed/strongly disagreed that the incentives had no impact on their PA (Table 4).

Although the sample size was small, survey responses indicated that incentives appeared to be more motivating for those with lower education levels. Of those with low education levels, 46% (6/13) compared to 13% (2/16) and 9% (3/34) with medium and high education levels respectively stated financial reasons as a reason to participate in *trips4health*. For intervention group participants with low levels of education, 86% (6/7) agreed/strongly agreed the value of the incentives motivated them to use the bus more, while 50% of those with medium (3/6; 50%) and high (8/16;50%) education levels agreed/strongly agreed. Interview participants (all medium/high levels of education) viewed incentives as potentially more motivating for those experiencing financial disadvantage although concessions for bus travel were acknowledged as a potential confounding factor:

*definitely low SES [socioeconomic status] and maybe that sort of band just above that where not everyone thinks catching the bus is that cheap. I mean, lots of people might get concessions or discounted [P113].*

For intervention participants 76% strongly agreed/agreed that the frequency of motivational text messages was just right, 90% strongly agreed/agreed that the content was easy to understand, and 48% strongly agreed/agreed that the messages were helpful (Table 4). 55% indicated that the text messages made no difference to their bus use and 55% indicated that they made no difference to their PA behaviours (Table 5). Interview participants discussed the generic nature of the messages and suggested that this compromised their impact: *the more individual you can get it, of course the more accountable I would have felt [P69].* Personalising the messages was suggested as a possible improvement:

*whether it would be worth trying a tact that was a bit more or less sort of just data driven and sort of more like one of your mates saying come on, let’s hop on the bus and go somewhere. I guess a bit more personal rather than just informative might be something that could work [P179].*

**Table 5:** Impact of text messages on bus use and physical activity behaviours, surveys *N* = 29

Behaviour	Response % (n)	Illustrative quotes about impact of text messages
**Bus Use**		*Well, I think the text messages, I found that for me, personally, were a little redundant because these were messages that I already knew. [P178]*. *I don’t think so. But I mean, it’s hard to know. Definitely not in that moment, whether subconsciously it helped me stay more aware. Overall, I don’t think so. [P247]* *It was a good reminder to do some physical activity, but it didn’t really affect my behaviour, I guess. It’s a good reminder [P88]* *I think it was already on my mind because of maybe the notifications about reaching targets and things. So, it wasn’t like it reminded me to do it or anything. [P69]*
The text messages made no difference to my bus use	55 (16)	
The text messages made me think about increasing my bus use	38 (11)	
The text messages made me increase my bus use	7 (2)	
**Physical Activity**		
The text messages made no difference to my PA	55 (16)	
The text messages made me think about increasing my PA	34 (10)	
The text messages made me increase my PA	10 (3)	

*PA* Physical acitivity

Participants in the intervention group also received weekly emails about their weekly bus trip target as part of administration of the intervention, with 59% of intervention participants strongly agreeing/agreeing that the weekly emails were helpful. Interviews revealed that the timing of these emails may have impacted their utility:

*I mean ideally it would be good, say on a Sunday night, to get a notification, “This week you have to catch the bus five times to reach your incentive,” because there are times when I thought, how many times am I needing to catch the bus? [P69].*

All *trips4health* participants received a copy of Australia’s Physical Activity and Sedentary Behaviour Guidelines with 78% reporting reading all or some of the guidelines. Those with high education levels (88%; 30/34) were more likely to read all or some of the guidelines compared to those with medium (68%, 11/16) and low education levels (61%; 8/13). Of the 49 participants who reported reading some or all the guidelines, 59% strongly agreed/agreed that it was helpful (Table 4). Interview participants who could recall reading the guidelines commented that *They did just reinforce why I wanted to do it, and the benefits. It was a good little reminder and prompt [P247]*. Others said that they were already aware of the information contained in the guidelines.

Administrative data showed from a total of 666 possible bus trip targets (based on total number of intervention participants and weeks of participation), 375 were met (56%) by 53 of 55 intervention group participants, with two participants not meeting any weekly targets (both had valid smartcards with one using the bus but not meeting targets and one withdrawing from *trips4health* because it was too burdensome). Of intervention participants, 38% strongly agreed/agreed that ‘Meeting the bus trip targets was easy’ with 41% disagreeing/strongly disagreeing (Table 4). Open ended survey responses and interviews provide some insight into the personal, social and system level barriers and enablers to meeting bus trip targets (Table 6). Lacking motivation, household commitments and limited bus availability were identified as barriers. Bus trip targets and incentives, sense of community and adequate bus availability enabled bus use.

**Table 6:** Illustrative quotes for barriers and facilitators for

Meeting bus trip targets	Barriers	Facilitators
Personal – targets and incentives	*I have struggled to increase my bus trips in recent weeks. My lifestyle is not conducive to manage the trips on a regular basis. [PS18]* *I tried to reach those targets. Like when I knew that I had this and this amount of number of times that I had to catch the bus, I tried to catch the bus anytime, even though it was hard sometimes.* [P88]	*it was in the back of my mind. I knew what the target was for the week, and you know, if I had the choice - do I get a lift today or do I catch the bus, and I knew I had to catch the bus just one more time to reach the target, it would drive me to, “Oh let’s just catch the bus.” [P69]* *if you know that you get paid, that you get an incentive, you know you will take the bus. If you wouldn’t get paid that amount if you’re taking the bus, you just take the car sometimes or more often as well [P88]* *I enjoy catching the bus, love the feeling of freedom it gives. I can walk for a bit if I feel like it or catch the bus. [PS377]* *Goal setting for greater use of the bus service [P363].*
Social	*I found the biggest and really the only factor as to whether or not I catch the bus to work is the actual requirements of my work. Sometimes I am required to have my car at work and use it for work and sometimes I am not [PS84]* *It was hard to drop the routine of driving as I drop my partner to work on my way to my own job [PS113]*	*Starting to feel like part of a new community when* *recognising familiar faces on rides. [PS179]* *I used to catch the bus to work quite regularly, but then since my [child] came along I haven’t been. So I went from zero to quite regular bus use. … Like take my [child] to school. At the time it was childcare, so it was a bus trip away, and going shopping. Just stuff that I would literally never, ever have gotten on a bus before. [P247]*
Service	*There are no buses in X so I cannot take a bus to [suburb] [PS196].* *I think people wouldn’t mind using the bus that often if the bus would be actually on time. Not just on time, like being delayed, but also leaving too early. Like it happened a couple of times that the bus just left even though I was there on time, [P88]* *But the route I’m on, …. they’re drinking alcohol on the bus and stuff … So, that’s why I’m saying I pick which buses to catch and at what times. [P413].*	*I worked with [company] and I had to [work] in and around Tasmania, I could use the bus to actually go to different places rather than wait for someone to take or to bring the car. That’s something that was really enjoyable. … And on the other side, basically I got to know all the stations because sometimes when you take a car, you don’t really know where you’re going, but when you’re taking your own buses, you know which stop to take. You have to walk a little bit, like look at the surroundings and that gives you a bit of exercise as well. I think the overall effect was quite positive. [P178]*

At study commencement, participants nominated their intended level of assessment participation which was categorised as: high, medium, or minimum (Table 7). At Timepoint 1 all participants intended to partake at the highest level, although 77% completed all possible baseline measures. The nominated, compared to actual assessment completion rates at Timepoint 2 were impacted by COVID-19 imposed restrictions for some participants as captured by administrative data.

**Table 7:** Nominated compared with actual data collection participation level (completed assessments) at Timepoint 1 and Timepoint 2

Data Collection Participation level^*^	T1		T2	
	Nominated	Actual	Nominated	Actual
High	110	85	52^‡^	40
Medium	0	13	9	17
Minimum	0	0	5	0
< Minimum	0	12	1	10
Withdrew/Lost to follow-up			5	6

*High: survey, smartcard data, accelerometer, travel app, physical measures; Medium: survey, smartcard data, accelerometer, and either but not both of app or physical measures (no app or physical measures but not both); Minimum: survey, smartcard data, accelerometer (no travel app, no physical measures); <Minimum: survey, accelerometer physical measures, travel diary OR used travel app, but not survey, smartcard data AND accelerometer. ^‡^ 2/52 nominated high T2 participation level but said could not attend clinic visit. App use: used app at T1 or T2

## Discussion

*trips4health* was designed to investigate the impact of financial incentives (bus trip credits) on bus trip use for PA gain. A process evaluation of *trips4health* was conducted to assess the key elements of reach, fidelity, feasibility and acceptability. The *trips4health* process evaluation demonstrated good acceptability regarding using financial incentives to increase bus use and strong fidelity and feasibility. The process evaluation exposed that even with a financial incentive and supporting theory-informed motivational techniques (text messaging), the desired behaviour change (increased bus trips) was not easily achieved, with just over half the possible bus trip targets met across all intervention participants. Due to early cessation of *trips4health* because of COVID-19, data were unavailable on the longer-term impact of financial incentives on bus use and PA.

While demographic characteristics of intervention and control participations were similar, the evaluation found limited reach for a population-based study, with a disproportionate number of participants being tertiary educated and female. Some recruitment strategies were conducted through university and government agencies which may have contributed to the higher number of participants with a tertiary level education. It is possible that bus use eligibility criteria (using the bus less than or equal to two trips per week in the past 6 months) may have limited the reach to those without a tertiary education because bus use is influenced by socioeconomic factors, although the association between socioeconomic status and transport behaviour is complex [24]. Furthermore, the over-representation of females in *trips4health* may be because females are more likely to use public transport compared with males, according to a recent analysis of travel surveys from 19 cities in 13 low, middle and high income countries, including Australia [25].

Recruiting harder to reach groups commonly requires additional resources, targeted strategies and community partnerships to be effective [26]. Future studies may need to adopt more targeted recruitment strategies to ensure appropriate representation of population groups less likely to engage in a study of this type. The under-representation of males and those without a tertiary education in *trips4health* makes it difficult to make recommendations with respect to adopting financial incentives as a population-wide strategy to increase bus use and subsequent PA.

Related studies have assessed the impact of incentive-based interventions on adult health behaviours [27, 28] including PA [9, 10], although none specifically on transport-related PA. Incentive-based strategies had been identified as likely/extremely likely to impact public transport use [28, 29] and were identified as a preferred strategy in formative work investigating acceptability of strategies to increase public transport use [30]. Analysis of bus use data for participants in this trial provide some evidence of effectiveness of incentives for increasing bus use (paper under review). However, qualitative data collected revealed that even with financial incentives, changing bus use behaviour is challenging. The inclusion of other proven behaviour change techniques (e.g. goal-setting, self-monitoring, social support) [31] via motivational text messaging did not fully address this issue. Participants indicated that being rewarded for reaching bus trip targets was motivating, but even with an increasing incentive scale and the supportive text messages, intervention participants found it difficult to attain the bus trip targets. This could be due to the *trips4health* intervention being focused on individual behaviour and personal motivations, whereas interviews and survey responses indicated that decisions about public transport use are influenced by broader social and environmental factors such as household needs, work requirements, and bus service characteristics that were beyond the control of individuals. It may also be that the bus trip targets were set too high. This could be tested in future studies.

### Strengths and limitations

To our knowledge *trips4health* is the first RCT to use incentive-based strategies to target transport-related PA. It was theory-informed and conducted in partnership with potential implementers. It had extensive process measures as well as a range of objective outcome measures. Unfortunately, *trips4health* was severely impacted by the COVID-19 pandemic which prevented recruitment of the anticipated sample size and long-term follow up of participants. The lack of long-term follow up prevented additional understanding of the impact of incentives on maintenance of public transport behaviours and retention in *trips4health* post the intervention phase. However, prior to study cessation there was sufficient data collected to evaluate fidelity, acceptability and reach, providing useful information for future iterations of this and related work.

## Conclusions

This process evaluation of *trips4health* provides evidence of the feasibility and acceptability of delivering a real-world intervention incentivising bus trip use for potential PA gain in partnership with a public transport provider and state and local government. Future studies investigating the use of public transport incentives to promote physical activity require collaborative relationships with the public transport provider to support smooth implementation of the incentive process, adoption of a range of recruitment strategies to ensure sufficient diversity among participants, include personalised behaviour change support strategies and ensure trip targets are achievable. While *trips4health* demonstrated good acceptability and support for incentivising public transport use, the process evaluation highlighted that individually targeted strategies may be insufficient to support travel-related PA behaviour change.

## Supplementary Information

**Additional file 1.**

## Data Availability

The datasets used and/or analysed during the current study are available from the corresponding author on reasonable request.

## Abbreviations

PA: Physical activity
RCT: Randomised controlled trial
