# Supplementary material for: Incentivising public transport use for physical activity gain: process evaluation of the COVID-19 disrupted trips4health randomised controlled trial
Source: Int J Behav Nutr Phys Act. 2022 Dec 22;19:157. doi: 10.1186/s12966-022-01394-x (PMC9772596; doi:10.1186/s12966-022-01394-x)
Supplement: Supplementary file 1 — Additional file 1. [file 12966_2022_1394_MOESM1_ESM.docx]

**Additional File Tables and figures: Process evaluation**

**Figure A1. *trips4health* Program logic***
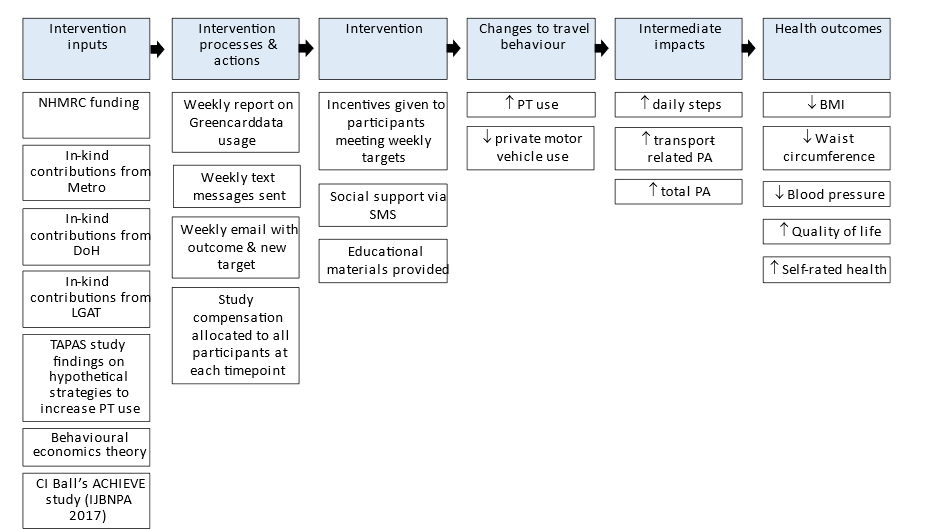
*

NHMRC = National Health and Medical Research Council, LGAT = Local Government Association Tasmania, DoH = Department of Health, TAPAS = Travel and Physical Activity Study, CI = chief investigator, SMS = short message service, Greencard = public transport smartcard, PT = public transport, PA = physical activity, BMI = body mass index

**Additional File Table A1. Inclusion and Exclusion criteria**

| **Inclusion criteria** | **Exclusion criteria** |
| --- | --- |
| - age 18+ years - sufficient English proficiency to provide informed consent (determined by ability to complete screening questionnaire and professional judgement) - making trips by car that could be made by bus - current infrequent bus user (≤ 2 trips week in the past six months) - possession of or willingness to possess a public transport smartcard - willingness for public transport provider and the researchers to access public transport smartcard data - living in Southern Tasmania - able to access an urban bus service - possession of a mobile phone | - intending to move house or work location whereby an urban bus service in Southern Tasmania can no longer be accessed within the 10-month study period - currently engaged in or planning to engage in other incentive based programs to enhance PT use - pregnant - health condition that prevents walking - health condition that prevents bus use - planned activity that would prevent using the bus for extended periods during the four-month intervention period (>2 weeks) e.g. surgery, extended holiday |

**Additional File Table A2. Bus trip targets**

| **Intervention timeline** | **Intervention phase** | **Target trips (n=above baseline)** | **Maximum weekly $ value of reward trips** ^a^ | **Text messages** |  |
| --- | --- | --- | --- | --- | --- |
| Week 1 | Getting started | 1 | 5.70 | x 2 |  |
| Week 2 | Getting started | 1 | 5.70 | x 2 |  |
| Week 3 | Slowly increasing | 2 | 11.40 | x 2 |  |
| Week 4 | Slowly increasing | 2 | 11.40 | x 2 |  |
| Week 5 | Moving along | 3 | 17.10 | x 2 |  |
| Week 6 | Moving along | 3 | 17.10 | x 2 |  |
| Week 7 | Moving along | 3 | 17.10 | x 2 |  |
| Week 8 | Moving along | 3 | 17.10 | x 2 |  |
| Week 9 | Aiming high | 4 | 22.80 | x 2 |  |
| Week 10 | Aiming high | 4 | 22.80 | x 2 |  |
| Week 11 | Aiming high | 4 | 22.80 | x 2 |  |
| Week 12 | Aiming high | 4 | 22.80* | x 2 |  |
| Week 13 | Maintenance | 5 | 28.50 | x 1 |  |
| Week 14 | Maintenance | 5 | 28.50 | x 1 |  |
| Week 15 | Maintenance | 5 | 28.50 | x 1 |  |
| Week 16 | Maintenance | 5 | 28.50 | x 1 |  |
| ***Total*** | | **54** | **$307.80** | **28** | |

*Incorrectly shown as 5 trips and 28.50 in information provided to participants.

**Additional File Table A3. Text messages**

| **When** | **Push notifications** | **Informed by qualitative data*** | **Behaviour change techniques^†^** |
| --- | --- | --- | --- |
|  |  |  |  |
| Week 1 | Hi X, XXX here. Welcome to Trips4Health! Today's the day to start working towards your goal of using the bus more to get more physical activity into your week. |  | Welcome  Social support |
| 1 | Hi X, XXX from Trips4Health here. Regular physical activity lowers your risk of heart disease, T2 diabetes, some cancers & more. Be more active by using the car less and the bus more! |  | Reward, repetition and substitution, natural consequences |
| 2 | Breaking physical activity into smaller sessions can help you fit it in and keep you healthy - walking to and from the bus stop is a great way to get small bouts of physical activity into your day. XXX (name of researcher) | ✓ | Reward  Goals and planning |
| 2 | Setting small goals can help motivate you. Why not try setting a goal to catch the bus 2 times more than usual this week? |  | Goals and planning |
| 3 | People who catch the bus do more physical activity than car users. Getting more physical activity into your week will make you healthier. Even a small amount of walking is good for you | ✓ | Natural consequences  Shaping knowledge |
| 3 | Don’t let the hills get in your way from walking - they're extra good for you. | ✓ | Reward  Shaping knowledge |
| 4 | Do you know how you are getting from A to B tomorrow? Think ahead about how you could use the bus to get more physical activity into your day. XXX |  | Goals and planning |
| 4 | Use the time on the bus to read, catch up on phone messages or prepare for a work meeting | ✓ | Reward |
| 5 | Have you revisited your bus use goals? Try setting a new goal this week and get more physical activity in whilst you are at it. XXX |  | Goals and planning |
| 5 | Remember you can get off a bus stop or two later to get in more physical activity - it will help you achieve the daily target of 10,000 steps | ✓ | Goals and planning  Natural consequences |
| 6 | Plan more walking into your bus trips this week by walking to or from a more distant bus stop. Catch an earlier bus so you have more time to walk to your destination. | ✓ | Goals and planning |
| 6 | Meet new people by catching the bus - there's often someone to talk to at the bus stop |  | Natural consequences |
| 7 | Have you ever used the [public transport provider] App to help plan your journey? Type Y or N XXX |  | Goals and planning |
| 7 | Catch an earlier bus so you have more time to walk to your destination. |  | Goals and planning |
| 8 | Well done! You are half-way through the Trips4Health program. Congratulations and stick with it! XXX |  | Social support  Feedback and monitoring |
| 9 | Schedule the days you will use the bus this week in your diary; wear comfortable shoes so that you can easily walk to or from a more distant bus stop. XXX |  | Goals and planning |
| 9 | Invite a friend or colleague to catch the bus with you - an easy way to catch up |  | Social support |
| 10 | Keep track of your transport behaviour and physical activity. Can you add more bus trips into your week to get more physical activity in? XXX |  | Feedback and monitoring  Goals and planning |
| 10 | Now that you have been catching the bus for a while, it shows that you can do this - keep going! |  | Self-belief |
| 11 | Try a different bus route this week that is a little bit further to walk to but may actually get you to your destination quicker. | ✓ | Goals and planning |
| 11 | Got some competitive friends? Why not see who can catch the bus the most in the next two weeks and allocate a prize to the winner |  | Reward  Social support  Comparison of behaviour |
| 12 | Ever thought about catching the bus with someone else? It's great for motivation. |  | Social Support  Shaping knowledge |
| 12 | Think about the good you are doing for the environment - by catching the bus, you are helping to reduce greenhouse gas emissions |  | Reward |
| 13 | Brisk walking is one of the simplest and cheapest forms of physical activity you can do almost anywhere - all you need are comfy shoes. Briskly walking to and from the bus stop is good for you XXX | ✓ | Reward  Shaping knowledge |
| 14 | Thinking about using the bus for a different journey but unsure of what bus to catch and where? The [public transport provider] App will give you all the information you need XXX | ✓ | Goals and planning  Shaping knowledge |
| 15 | Don't let bad weather stop you from taking the bus and being more active - rug up, wear a jacket and grab your brolly! XXX |  | Goals and planning  Self-belief |
| 16 | Congratulations, you've reached the final week of Trips4Health! We'll be in touch! Keep catching the bus. Its good for everyone. XXX |  | Social support |

* From studies conducted in 2017 and 2018 on transport behaviour and physical activity

^†^Based on Behaviour change techniques (Michie et al 2013)

**Additional File Table A4. Interview schedules**

| **Implementation partner interviews** |  |
| --- | --- |
|  | *Pre-intervention*   - What do you hope to gain from being involved in this research project? - How have you been preparing for the implementation of this study and the roll out of the incentive scheme? - Have you had to change systems and processes to accommodate this project? Can you elaborate or outline what changes you have had to make? - Will these changes be used/applied by your organisation in other ways? - What have been the greatest challenges for you in developing this incentive scheme? - If this project was successful, what might get in the way of this intervention being implemented more broadly at [public transport provider]? - Are there any policy or practice changes coming up that may impact the research project? - Can you tell me how you currently use the Greencard data? Is the use of this data for this study different to usual data processes? |
| **Intervention Participants** |  |
| **General** | Tell me about your involvement in the *trips4health* intervention…  Has anything changed for you because of your involvement with the *trips4health* study?  What impact did having public transport targets have on your public transport use? |
| **Greencard (smartcard) incentive** | What impact - if any - did the study incentive (i.e. Greencard credit) have on your public transport behaviours? Physical activity behaviours?  How did having an incentive impact your public transport behaviours?  What do think about the level/amount of the incentive provided in this study?  What did you think of the timing of the notification about reaching the targets and receiving the incentive? Did either of these things change your public transport use? |
| **Text Messages** | What impact – if any - did the text messages have on your public transport and physical activity?  How did the messages impact your behaviour and decisions to use public transport (or increase PA)?  What did you think about the content of the text messages?  Were there any particular messages that you liked more than others?  What did you think about the frequency/timing of the text messaging? |
| **Written Materials** | What did you do with the written materials provided for this study (e.g. read, think, act)?  What impact did the written materials have on your public transport (and PA) behaviour? |
| **Wrap up** | What impact do you think having an incentive scheme to increase PT use might have on all Tasmanians?  Any other comments to make? |

**Additional File Table A5. Issues identified from pilot study participants (survey n=5, verbal feedback to research staff n=11) and research staff administering the pilot study and corrective action taken**

| *Topic* | *Tool/ Assessment* | *Findings* | *Corrective action* |
| --- | --- | --- | --- |
| Recruitment | Screening survey and recruitment communication | - Signing up for the study was easy (5/5) - Mixed views on whether there was too much (2/5)/ or the right amount (3/5) of information provided in recruitment communication | No |
| Baseline measures | Clinic assessment | - All survey participants found the clinic assessments easy to attend - 1/5 suggested adding duration of clinic visit to information materials | No |
|  | Accelerometer | - Easy to use, but some participants were unsure if the accelerometer was working because the device provides no feedback (1/5 in survey; also verbally reported) - Some found the accelerometer initially uncomfortable (2/5) | No |
|  | Travel app | - 2/5 found the app difficult to use, 2/5 easy to use, 1/5 didn’t use - Most reported that either the app did not record trips (1/4; also verbally reported), or that the app incorrectly recorded trips (3/4) - Some confusion about what counted as a “trip” | - Extensive refinements of app by app provider with ongoing testing - Paper-based travel diary included as a data collection method in case of app errors during study period. - Example trips added to diary |
|  | Survey | - 3/5 easy to complete, 2/5 difficult to complete. - Generally, participants thought survey was longer than expected and 1/5 commented that it was repetitive in parts | Text added at beginning of survey providing detail about approximate completion time, question context, justification for question inclusion and how survey questions may be analysed |
| Intervention | Text messages | - 4/5 reported that the text messages were useful - 1/5 was unaware that text messages were part of the intervention. Participant preferred email and wanted an an option to opt out of text messages | At randomisation research staff told participants that weekly text messaging was part of the intervention. |
|  | Incentives | Smartcard credits were not issued in the pilot study | N/A |
|  | Intervention group pamphlet | 4/5 found the pamphlet easy to understand, but some found the rewards table confusing (1/5; also reported verbally) | Table in pamphlet edited for clarity |
| Communication | Phone/email communication with research team | Easy and responsive (5/5). | N/A |
| Other |  | - Some confusion about the distinction between the app and the accelerometer amongst pilot study participants which led some to stop using the accelerometer if the app malfunctioned - Research staff identified smartcard number entry errors - Research staff identified opportunity to improve communication to participants about whether bus trip targets had been met - Public transport provider determined opportunity to refine process for identifying participants meeting bus trip targets | - Accelerometer and app instructions and app FAQs updated to clarify the difference and independence between the two data collection methods - Editable field set up in database to enable checking and correction of smartcard details - Improved communication to participants about bus trip targets being reached or not - Database updated to simplify process for reporting to public transport provider those participants meeting bus trip targets |

**Additional File Table A6. Issues identified from audit and anecdotally from participants and corrective actions taken**

| Issue | Action |
| --- | --- |
| Some participants not given current Participant Information Sheet and Consent Form (PICF) | - Correct PICF provided to and signed by respective participants. - Improved record keeping of PICF version and PICF completion by participants in databse. |
| Errors in incentive calculation database:   - Database did not originally multiply incentive payment by bus target trips - At week 12, bus trip target incorrectly documented as 5 trips and should have been 4 trips | - Formula to calculate incentives corrected - Back payment of smartcard credit issued to affected participants |
| Several app user-related issues identified by participants | Several corrections applied to the app, with the latest applied 27-28 Feb 2020. |
| App overwrote inferred trip type (i.e. app inferred type of transport behaviour such as walking, driving) with categorized trip type (participant confirmed/refuted inferred trip type) when both were required for the study | App provider corrected the error for future use but could not correct past over-writing errors |
| Confusion about clinic or survey requirements | Editing of information disseminated with clinic or survey invitations |

**Additional File Table A7. Response to recruitment methods**

| **Recruitment method** | Social  media | Traditional media ^†^ | Bus advertising | Word of mouth | Professional networks ^‡^ | Direct  email | Other ^§^ | No  answer |
| --- | --- | --- | --- | --- | --- | --- | --- | --- |
| By total enquiring about the study (n = 912) | 14 % (131) | 5 % (45) | 17 % (159) | 3 % (23) | 5 % (48) | 3 % (28) | 1 % (10) | 51 % (468) |
| By total who disclosed recruitment method (n = 444) | 30 %  (131) | 10%  (45) | 36%  (159) | 5% (23) | 11%  (48) | 6%  (28) | 2%  (10) | - |
| By study participants (n = 110) | 35% (38) | 12% (13) | 24% (26) | 6% (7) | 11% (12) | 10% (11) | 2% (2) | 1% (1) |

† radio, television, print (includes flyers, except bus advertising), billboards ‡ workplace intranet, newsletters or websites § did not describe, received information through another study, browsing the internet, could not recall

**Additional File Table A8. Reasons for ineligibility and total number ineligible and consented**

| **Reason for ineligibility** | **n** |
| --- | --- |
| Unable to access Southern Tasmanian urban bus service during study period (next 10 months) * | 32 |
| Planning to/engaged in other public transport use incentive program | 22 |
| Pregnancy | 9 |
| Health condition preventing walking | 9 |
| Health condition preventing bus use | 8 |
| Planned activity preventing bus use for >2 weeks during intervention phase (e.g. surgery) | 33 |
| Under 18 years | 1 |
| Catches bus more than twice a week | 120 |
| Does not make any trips by car that could be made by bus | 106 |
| Not willing to get a Greencard and give consent to analyse Greencard usage | 11 |
|  |  |
| No mobile phone | 3 |
| Ineligible | 201 (78 of whom fully completed/submitted screening survey) |
| Eligible | 243 (228 of whom fully completed/submitted screening survey) |
| Interested in participating | 221 |
| Consented | 184 |

* Includes answering “No” to (1) Do you live in Southern Tasmania? Or (2) Can you access a bus? Or answering “Yes” to (1) Are you intending to move house or change your work location whereby you will no longer be able to access an urban bus service in Southern Tasmania during the study period (the next 10 months)?
